# Green Carbon Dots: Synthesis, Characterization, Properties and Biomedical Applications

**DOI:** 10.3390/jfb14010027

**Published:** 2023-01-02

**Authors:** Hong Hui Jing, Fevzi Bardakci, Sinan Akgöl, Kevser Kusat, Mohd Adnan, Mohammad Jahoor Alam, Reena Gupta, Sumaira Sahreen, Yeng Chen, Subash C. B. Gopinath, Sreenivasan Sasidharan

**Affiliations:** 1 Institute for Research in Molecular Medicine (INFORMM), Universiti Sains Malaysia (USM), Pulau Pinang 11800, Malaysia; 2 Department of Biology, College of Science, University of Hail, Hail P.O. Box 2440, Saudi Arabia; 3 Molecular Diagnostics and Personalized Therapeutics Unit, University of Hail, Hail P.O. Box 2440, Saudi Arabia; 4 Department of Biochemistry, Faculty of Science, Ege University, Izmir 35040, Turkey; 5 Nanotechnology Research and Application Center, Sabanci University, Istanbul 34956, Turkey; 6 Department of Chemistry, Faculty of Science, DokuzEylül University, Izmir 35390, Turkey; 7 Department of Pharmacognosy, Institute of Pharmaceutical Research, GLA University, Mathura 281406, India; 8 Department of Oral & Craniofacial Sciences, Faculty of Dentistry, University of Malaya, Kuala Lumpur 50603, Malaysia; 9 Faculty of Chemical Engineering and Technology, Universiti Malaysia Perlis, Arau 02600, Malaysia

**Keywords:** carbon dots, natural resources, green synthesis, fluorescent nanoparticles, biomedicine, biomedical applications

## Abstract

Carbon dots (CDs) are a new category of crystalline, quasi-spherical fluorescence, “zero-dimensional” carbon nanomaterials with a spatial size between 1 nm to 10 nm and have gained widespread attention in recent years. Green CDs are carbon dots synthesised from renewable biomass such as agro-waste, plants or medicinal plants and other organic biomaterials. Plant-mediated synthesis of CDs is a green chemistry approach that connects nanotechnology with the green synthesis of CDs. Notably, CDs made with green technology are economical and far superior to those manufactured with physicochemical methods due to their exclusive benefits, such as being affordable, having high stability, having a simple protocol, and being safer and eco-benign. Green CDs can be synthesized by using ultrasonic strategy, chemical oxidation, carbonization, solvothermal and hydrothermal processes, and microwave irradiation using various plant-based organic resources. CDs made by green technology have diverse applications in biomedical fields such as bioimaging, biosensing and nanomedicine, which are ascribed to their unique properties, including excellent luminescence effect, strong stability and good biocompatibility. This review mainly focuses on green CDs synthesis, characterization techniques, beneficial properties of plant resource-based green CDs and their biomedical applications. This review article also looks at the research gaps and future research directions for the continuous deepening of the exploration of green CDs.

## 1. Introduction

CDs, one of the essential fluorescent carbon nanoparticles, are earning increasing consideration in everyday human life for their practical applications. Generally speaking, CDs are a new category of zero-dimensional carbon-based nanoparticles that can be allocated into three groups according to their structural manner: CDs, carbon quantum dots (CQDs) and graphene quantum dots (GQDs) [1]. CDs are amorphous quasi-spherical nanoparticles with no quantum confinement effect (QCE), in which the band gaps are independent of their sizes; CQDs are comprised of crystalline, quasi-spherical fluorescent carbon nanomaterials with a diameter between 1 nm and 10 nm and possess a moderate QCE, where the band gap is influenced by the CQD size; GQDs are carbon nanoparticles that consist of several layers of zero-dimensional graphene sheets with sizes between 1 nm and 10 nm that exhibit strong QCE, where their band gaps strongly rely on their sizes [1]. CDs (firstly identified as “carbon nanoparticles”) were accidentally founded by Xu et al. [2] in 2004 during the separation and purification of single-walled carbon nanotubes (SWCNTs) and triggered subsequent studies in both technological and scientific fields. This invention also attracted significant attention in discovering CDs’ fluorescence behaviours, leading to developments in the field of CD research. The discovery of CDs also attracted the interest of various researchers to explore the structurally related cousins of CQDs (e.g., carbonized polymer dots (CPDs)), which are characterized based on their structural differences, formation mechanism and properties [3]. In 2006, these newly discovered fluorescent carbon nanomaterials were titled “carbon quantum dots” by Sun et al. [4], who successfully disclosed a synthetic track to develop CQDs with much-enhanced fluorescence emission [5]. 

Recently, CDs have acquired significance in nano-chemistry due to their unique physical characteristics, especially their highly luminescent properties. CDs are akin to inorganic fluorescent semiconductor nanoparticles and have intensive tunable fluorescence characteristics [6,7]. Thus, CDs with this feature property provide insights for researchers to determine advanced applications and products related to CDs in various regions, especially in biomedical fields such as disease detection, drug delivery, bioimaging, biosensing, photocatalysis, electrochemical luminescence, therapeutic genes, photosensitizers and optronics [7,8]. CDs are made of carbon, a type of element that is abundant, non-toxic, and one of the examples of the building blocks of life itself. To date, several approaches have been applied to the production of CDs. However, green CDs synthesised from natural green resources have attracted the interest of scientists worldwide. Green CDs are carbon dots synthesised from renewable green sources such as biomass or renewable natural products, including vegetables, fruits, agro-waste and human derivatives. Natural green resources are an excellent source for synthesising CDs because they are affordable, easy to obtain, have high stability, have a simple protocol, are safer and are eco-friendly, with abundant carbon sources. In addition, the green CDs also express eco-friendliness compared to CDs derived from chemical precursors. The green synthesis of CDs has become the predominant sustainable green approach due to their valuable transformation of low-value bio waste into value-added products. These exclusive benefits also enable green CD applications in physical, chemical and biological fields, i.e., photonic device manufacturing, biological imaging and solar cells [6]. Moreover, green synthesised CDs also possess numerous beneficial characteristics, including tiny size, excellent water solubility, highly tunable photoluminescence (PL), biocompatibility, ease of modification, low toxicity, multi-photon excitation (up-conversion), economic scale-up production, unique fluorescence behaviour, electrochemiluminescence and flexibility in combination with other nanoparticles in comparison with organic dyes as well as traditional semiconductor quantum dots [7,8]. These valuable properties make green CDs easily conjugated with biomolecules. They are chemically inert and less toxic than carbon, which is commonly a black material with low solubility in water and weak fluorescence. Hence, CDs have become preferable to utilize as an effective carrier for drug delivery and biological imaging [7]. The electrochemical luminescence (ECL) and chemical luminescence properties of CDs are mainly attributed to their excellent electronic characteristics (e.g., CDs as electron acceptors or donors, which enable applications in the area of catalysis, optronics and sensors [9]). CDs are also applicable in industries like solar cells and light-emitting diodes [10,11]. In addition, CDs are combined with commercial inks and reserve their fluorescence properties in the solid state to be used as an anti-fraud agent for object identification, supermarket labelling, wearable optoelectronics and military security [9,12]. This review will emphasize the synthesis and characterization techniques of CDs from natural resources, summarise their properties, and underline their recent biomedical applications. 

## 2. Synthesis of Carbon Dots from Natural Resources

**Figure 1:**
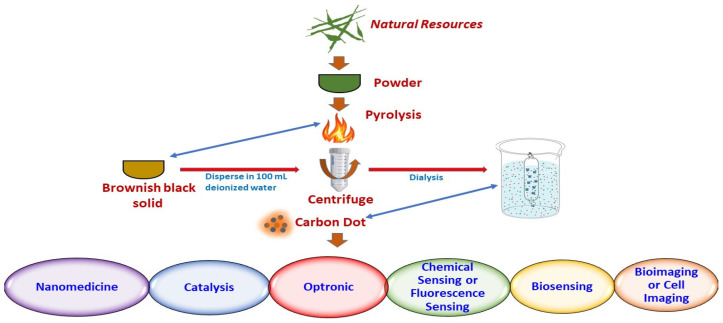
Carbon dot production from natural resources for various applications.

For the last decennary, many techniques have been discovered to develop CDs; these are ordinarily classified into two different manners, the “top-down” and “bottom-up” approaches for various applications (Figure 1). The “top-down” method specifies the preparation of CDs by fragmentation of big carbon precursor molecules into nanoscale particles, whereas the “bottom-up” method specifies the transformation of CDs from suitable molecular precursors under certain conditions [13]. These approaches can be achieved by chemical, electrochemical and physical methods, including modification functionalization, nanohybrids and doping during the preparation or post-treatment of carbon nanodots [14]. The yield of CDs could be improved using post-treatment or during arrangement. The modification of CDs is necessary to achieve benign surface characteristics, which are decisive for their solvency and operation [15]. Wang and Hu [14] summarised the development of CDs through numerous approaches, such as ultrasonic treatment, chemical oxidation, microwave irradiation, solvothermal techniques and hydrothermal techniques. Natural products such as ground coffee, fruits, vegetables, light sediment, tea leaves, agro-wastes and organic waste products, which are abundant in nature, can be utilized as carbon precursors to synthesize CDs [7]. These green preparation methods are greatly supported as they are green and economical as compared to chemical and physical actions. In addition, the conversion of waste materials into useful and valuable products is efficient to address environmental issues. Moreover, active carbon, graphite, nanodiamonds and carbon nanotubes can break down into CQDs via laser ablation, electrochemical techniques and arc discharge.

Synthesis of carbon dots from natural resources, such as plant-based carbon dots, is vital since carbon dots are useful in various health and electronic technologies and also to avoid producing this tiny nanomaterial from harmful and costly resources like metals. Moreover, building carbon dots from natural resources can also play a pivotal role in environmental pollution control by providing a new direction for developing ecological pollution control. Agro-waste usage for producing carbon dots also helps to reduce agro-waste-related environmental issues by reducing the vast environmental problem when agro-waste is left on plantation areas in large amounts. Various nontoxic medicinal plants have exhibited good in vitro and in vivo health-related biological activity against multiple ailments. However, a plant extract can seldom be used as a final product. Subject to the extraction method, plant extracts can contain various phytochemicals.

In many cases, from a pharmacological point of view, it is interesting to work with crude extract or fractions instead of a single isolated compound. This could be due to the multi-targeting effect of the various phytochemicals present in the plant extract. However, lack of knowledge of the active compounds, the synergistic effect of the extract compounds, poor stability, solvent toxicity, and low solubility of the bioactive compound must be overcome to achieve a final product or develop new products from medicinal plants. Interestingly, many nanotechnology-based strategies have recently been proposed as an alternative to solve these problems. Using this medicinal plant with good biological activity to develop new nanotechnology-based products, such as novel carbon dots, using nanotechnological approaches is highly recommended. Once fully developed, these green synthesised carbon dots will be the only readily accessible final product from medicinal plants, commonly reported to exhibit beneficial biological and pharmacological activities. Furthermore, green synthesised carbon dots could eliminate reliance on traditional medical practitioners or medicinal plantations for healing purposes. 

In addition, selecting particular agro-waste and medicinal plants for green synthesised carbon dots is economically viable since this approach can increase farmers’ income and support the agriculture industry. Moreover, non-dimensional fluorescent semiconductor dots are valuable for various electronic technologies as they are made of nontoxic, inexpensive natural resources like plants. These unique features focus on health benefits and environmental problems, such as converting agro-waste into valuable products and producing nontoxic fluorescent semiconductor dots for various electronic technologies by using green synthesis techniques to produce carbon dots, indicating the importance of green synthesis of carbon dots from natural resources. 

### 2.1. Green CDs Synthesis via “Top-Down” Approaches

**Figure 2:**
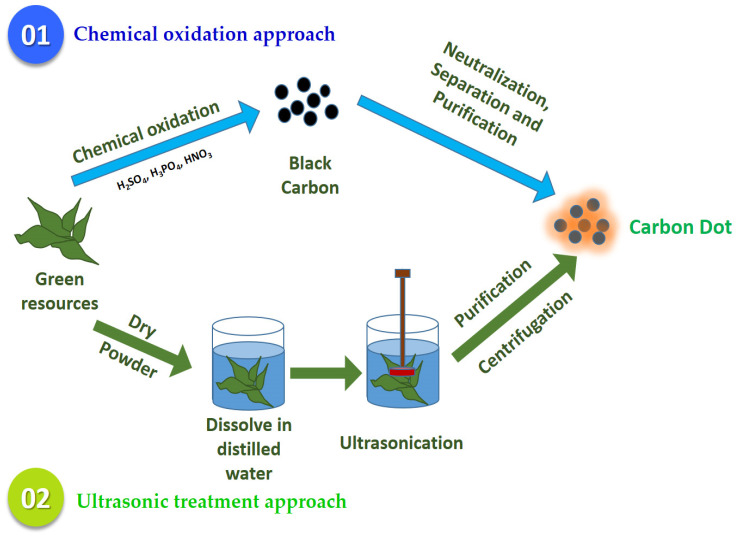
Top-down approaches in green synthesis of carbon dots.

As mentioned above, “top-down” approaches refer to the breakdown of carbon materials into CDs. These “top-down” strategies comprise chemical oxidation and ultrasonic treatment [13,16] (Figure 2).

#### 2.1.1. Chemical Oxidation Approach 

Chemical oxidation is one of the most familiar “top-down” approaches for the preparation of CDs. This is recommended by various researchers due to their remarkable advantages, including low-cost, large production yield, high purity and easy size control [13]. In chemical oxidation methods, small organic molecules are carbonized to carbonaceous materials by using strong oxidizing acids [14]. Typically, CDs prepared by this technique are usually abundant in functional groups, which gives them more extensive applications in the sensor [17]. Peng and Travas-Sejdic [18] expressed an easy way to prepare luminescent CQDs via dehydration of carbohydrates using concentrated H_3_PO_2_. The carbonaceous materials produced were then treated with HNO_3_ to break down into individual CQDs followed by passivation processes using amine-terminated compounds yielding luminescent CQDs [18]. Gunja et al. [19] chose HNO_3_ as an oxidant and waste tea residues (WTR) as the carbon sources to produce eco-friendly CDs. The WTR was first dried and ground into fine powder. Next, the obtained powder was dissolved in HNO_3_ and carbonized for 6 h. The obtained mixtures were then centrifuged, neutralized with Na_2_CO_3_, filtrated and dialyzed against pure water for 24 h to obtain fine CDs [19]. Similarly, studies also reported that H_2_SO_4_ and H_3_PO_2_ were applied as an oxidant to prepare CDs from natural sources such as tomato [20], muskmelon fruit [21] and green tea leaf residues [22]. However, the use of strong oxidizing acid in the preparation of CQDs is hazardous for the environment and often results in the formation of toxic gas as well as many impurities deposited in the sample mixture. Additional steps are required to remove the excess acid and impurities, which is costly and limits the application of chemical oxidation approaches in CQD synthesis. Interestingly, Hu et al. [23] and Saikia et al. [24] proposed the application of H_2_O_2_ as an alternative oxidant rather than oxidizing acid to prepare CDs from coals. The existing research on the preparation of CDs from different carbon precursors by chemical oxidation approaches is shown in Table 1. It is well known that CDs synthesized by chemical oxidation approaches show tremendous photoluminescence, despite the potential chemical toxicity that should be taken into consideration by researchers [17]. As such, more attention should be given to solving this issue in any further studies.

#### 2.1.2. Ultrasonic Treatment Approaches

Ultrasonic treatment is one of the “top-down” approaches. It applies high-energy ultrasonic sound waves, able to break down huge carbon molecules into smaller CDs particles [13]. This method has been recommended due to its unprecedented benefits, such as being environmentally friendly, low-cost, and having strong penetration and a uniform effect [17]. For instance, Dehvari et al. [25] utilized crab shells to synthesize CDs. In this experiment, the crab shells collected were dried, ground into powder form and dissolved in water for ultrasonication irradiation. The solution was then filtered and centrifuged to obtain highly fluorescent CDs with a quantum field (QY) of 14.5% [25]. A recent experiment conducted by Zaib’s team used a simple procedure to prepare CDs. In this experiment, they dissolved dried *Polyalthia longifolia* leaf powder in distilled water and ultrasonicated the mixture for 1 h followed by centrifugation [26]. Lin et al. [17] expressed the synthesis of CQDs using simple carbonization approaches with varying surface morphology and large particle size, where ultrasonic synthesis could overcome these shortcomings. As such, the combination of ultrasound treatment with other methods is recommended to synthesize CQDs with excellent performance. For example, ReddyPrassad and Naidoo [27] pioneered the use of dextrose to produce CDs via an easy ultrasonic approach. The high fluorescence CDs obtained showed a significant increase in the photocatalytic efficiency when modified with copper tungstate (CuWO4), which has shown potential application in wastewater treatment [27]. Huang’s group also revealed that CDs that were prepared from cigarette ash via ultrasonication showed bright green fluorescence under UV light when functionalized with poly(ethylene oxide) [28]. Nonetheless, the major challenge for CD preparation via ultrasonic treatment is uneven heating of samples, which is mainly associated with the local thermal effect of ultrasonic waves that affects the efficiency of the reaction when compared to direct heating or microwave [17,29]. With these identified challenges, further investigations are needed to improve CD synthesis by ultrasonic treatment. Table 2 shows some of the current research that utilizes the ultrasonic method to prepare carbon nanodots. 

**Table 1:** The summary of CDs synthesized from different natural products by chemical oxidation techniques and the respective applications.

Carbon Source	Oxidising Agent	Application Field	References
Anthracite coal	H_2_O_2_	Pollutant control	[23]
Lignite coal	O_3_	Fluorescence sensor	[30]
Palm shell powder	CF_3_COOH	Fluorescence sensor	[31]
Muskmelon fruit	H_2_SO_4_ H_3_PO_4_	Biosensor	[21]
Waste tea residue	HNO_3_	Fluorescence sensor	[19]
Tomato	H_2_SO_4_H_3_PO_4_	Fluorescence sensor and bioimaging	[20]
Pennsylvania anthracite and Kentucky Bituminous coal	H_2_O_2_	NA	[24]
Green tea leaves	H_2_SO_4_	Fluorescence sensor	[22]

### 2.2. Green CD Synthesis via “Bottom-Up” Approaches

The “bottom-up” strategies for CDs synthesis are highly recommended by many researchers due to simple procedures, low cost, being environmentally friendly, inexpensive scale-up production and precise controllable design of initial molecules [34].

#### 2.2.1. Carbonization or Pyrolysis Synthesis

Carbonization or pyrolysis synthesis is recognized as the most common “bottom-up” method and was first proposed by Xu et al. [2] during the discovery of CDs; it has been applied by many researchers. According to Speight [35], carbonization is a process for the production of carbonaceous residue by the thermal decomposition of carbon-containing materials, particularly natural products. It is an inexpensive and eco-friendly method that requires high temperatures to decompose a compound [35]. Numerous studies have reported that the CDs can be prepared by carbonization of various food items and waste, including watermelon peels, Lychee seed, wool, lemon juice and others; see Table 3. For example, Zhou et al. [36] produced high-quality fluorescent CDs, synthesized using watermelon peel through a low-temperature carbonization filtration method. This was done by carbonizing the watermelon peel at 220 °C for 2 h in air, followed by sonication, filtration and centrifugation of products to produce CD solutions. This method is simple and the use of food waste (watermelon peel) as a novel carbon precursor is eco-friendly and economical and leads to the production of large-scale aqueous CDs without any post-treatment modification [36]. Another study conducted by Wei’s group successfully prepared CDs with strong blue light from gynostemma via the carbonization process. The carbonization process was performed by heating dried gynostemma in a porcelain boat at a temperature of 400 °C for 4 h in a N_2_ atmosphere. The carbonized sample was then dissolved in ultrapure water to produce a black solution. Filtration was then performed to remove large particles, and CQDs with diameters of 0.4–2.0 nm were finally obtained [37]. Interestingly, the CDs obtained were stable, had low biological toxicity and were able to promote the expression of mRNA of antioxidant-related genes of zebrafish [37]. Although pyrolysis is a simple way of producing CDs, challenges such as low QY of CDs and high requirements for equipment urged the researchers to explore a better solution for future development [17]. 

#### 2.2.2. Hydrothermal Carbonization and Solvothermal Carbonization Synthesis

Hydrothermal carbonization and solvothermal carbonization are recognized as other “bottom-up” approaches that require high temperatures and pressure to synthesize carbon-based materials (Table 4). They are widely used and loved by numerous researchers, as they are green, low in cost, non-toxic and require simple operation [14]. In a typical approach, De and Karak [52] synthesized CDs using the solvothermal route by heating banana juice in a glass bottle. The banana was first cut into small pieces and mixed with water to form a banana paste. Then, the banana paste was mixed with ethanol in a bottle and heated at 150 °C for 4 h in an oven to produce a black product. The product was dissolved in water, filtered and mixed with ethanol before the centrifugation process was performed to separate large particles. The supernatant collected was evaporated at room temperature to produce CQDs [52]. A similar experiment was conducted by Zhao et al. [53], who utilized corn bract to synthesize CDs. Additionally, the hydrothermal route utilizes natural products such as pomelo peels, tomato juice, leaves, olive pits, chitin and bitter melon as starting materials, placed in an airtight container with water under high pressure and temperature to produce CQDs [54,55,56,57,58,59]. Up to now, many studies have applied hydrothermal methods to synthesize CDs; see Table 4. For example, Lu et al. [54] demonstrated the employment of pomelo peel as a carbon precursor to obtain fluorescence CDs with a QY of approximately 6.9%. In this case, pomelo peels were mixed with water, poured into a Teflon-lined stainless steel autoclave and heated at 200 °C for 3 h. The CD mixture was then centrifuged to remove large particles. Interestingly, the Hg^2+^ was able to induce fluorescence quenching of CDs, which showed great potential to apply in Hg^2+^ detection, as the isolated CDs exhibited excellent selectivity and sensitivity towards Hg^2+^ [54]. Similarly, Yadav’s team utilized *Azadiracthta indica* leaves (neem leaves) as carbon precursors to isolate CQDs. The CQDs produced exhibit higher fluorescence QY yield, up to 27.2% [60]. The difference in QY was mainly due to the difference in the size of particles, types of functional groups present on the surface of particles and passivation techniques, where CDs isolated from different natural carbon precursors also showed different luminescence properties [17]. Liu et al. [59] synthesized CDs using bitter melon via simple and inexpensive hydrothermal approaches. They found that the synthesized CDs showed excellent sensing properties, as they were able to detect p-Aminoazobenzene (pAAB) effectively. CDs that are isolated from these natural products possess many surface functional groups, for example, hydroxyl, carboxyl and amino groups, without performing any chemical adjustment, yielding great hydrophilia [61]. Hence, hydrothermal approaches have turned into the primary approaches to preparing CDs due to their simple preparation procedures and low experimental requirements, as well as ease to achieve high QY [17,34]. However, the preparation of CDs by solvothermal and hydrothermal techniques in the production of CQDs with varying surface morphology and less controllable size have led researchers to explore possible solutions to overcome this shortcoming. 

#### 2.2.3. Microwave Irradiation

Microwave irradiation of organic compounds is a low-time-consumption, green, easy-temperature-control and low-cost method that is favoured by many researchers for synthesizing carbon nanoparticles. The production of CDs can be done by exposing the reaction mixture that contains carbon precursors under electromagnetic radiations with a wavelength ranging from 1 mm to 1 m [13]. Numerous studies have utilised this technique to synthesize CDs by applying natural products as carbon precursors; see Table 5. For example, fresh banana peels were mixed with water and transferred to a 500 W microwave and heated for 20 min. The mixture was then centrifuged, filtered and dialyzed to produce high QY CDs [92]. Furthermore, Ramezani, Qorbanpour and Rahbar [93] used quince fruit as a carbon precursor and distilled water as a solvent to synthesize CDs. In this research, the quince fruit powder was dissolved in water, transferred to the Teflon microwave vessel, sealed tightly and placed in a microwave oven for microwave irradiation treatment at a temperature of 220 °C for 30 min to produce CQDs. The as-prepared CQDs did not show any cytotoxic effect towards HT-29 cells and showed the potential of fluorometric detection of As^3+^ ions, which further reflected the potential application of CQDs in cell imaging, chemical sensing and drug delivery [93]. However, the quantum yield of CDs obtained from microwave irradiation is usually low (i.e., in Ramezani’s experiment, a QY of 8.55% was obtained) when compared to other methods. To overcome this shortcoming, the combination of microwave irradiation reaction with other methods such as pyrolysis is recommended to isolate CDs from natural products. In a typical synthesis, Gul and colleagues reported the preparation of CDs from banana peels via carbonization followed by microwave irradiation [51]. In this experiment, banana peels were carbonized at 80 °C for 12 h in an oven and ground to a fine powder. Then, the dried banana peel powder was dispersed in water and placed in a microwave oven for microwave irradiation for 5 min. Filtration and centrifugation were performed to remove large particles to produce high QY CDs [51]. 

## 3. Characterization of Carbon Dots

As a new member of the “nanoparticles universe”, the characteristic of CQDs has been broadly studied via various analytical techniques. Characterization of CQDs is necessary to attain a better understanding of the mechanism correlated with its exclusive physical properties. Diverse spectroscopic methods, including Fourier-transform infrared spectroscopy (FTIR) and ultraviolet-visible (UV-vis) spectroscopy, have been described for CD characterization. Transmission electron microscope (TEM), high-resolution electron microscope (HRTEM), zeta potential, quantum yield analysis and X-ray diffusion (XRD) are also recommended to characterize the isolated CDs from natural products. 

### 3.1. UV-Vis Spectroscopy Technique

**Figure 3:**
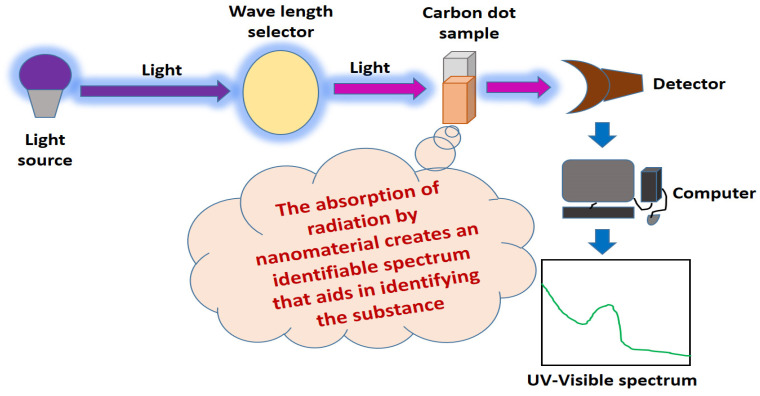
The basic setup for the measurement of UV-visible spectra.

UV-vis spectroscopy is usually recommended to evaluate the optical properties of CQDs, as the CQDs synthesized using various methods usually possess strong UV absorption yet result in a difference in absorption peaks [15]. The principle of UV-Visible spectroscopy is based on the absorption of ultraviolet light or visible light by chemical compounds, which results in the production of distinct spectra. Spectroscopy is based on the interaction between light and matter. When the substance absorbs the light, it undergoes excitation and de-excitation, resulting in the production of a spectrum (Figure 3).

The fractionation of C-dots was performed by high-performance liquid chromatography and gel electrophoresis to resolve CDs with different sizes and shapes and reported that the CDs with sizes of 1.2, 1.5–3 and 3.8 nm emit at the visible (400–700), UV (350 nm) and near-infrared (NIR) regions, respectively [15]. Hence, an absorption band peak centred on the UV region of 250–300 nm, which is also known as a typical π-π* transition peak, is common in most CDs. This was addressed by an experiment reported by Sun et al. [72], which presented that the CDs (diameter ~3.3 nm) synthesized from *Lycii fructus* through hydrothermal treatment emitted a strong absorption peak at 271 nm on the UV region. Murugan and Sundramoorthy [48] also reported that the UV absorption peak of CDs that were synthesized from *Borassus flabellifer* flower by thermal pyrolysis was 282 nm in the absorption region, which was ascribed to the π-π* transition of C=C bonds of aromatic rings [48]. Another study conducted by Shekarbeygi’s team also demonstrated that the CDs isolated from rose flowers via hydrothermal treatment had strong absorption peaks at 270 nm and 360 nm, which were also attributed to the π-π* transition of C=C bonds and 𝑛→𝜋* transition of C=O bonds in CDs. Interestingly, Shekarbeygi’s team found that the absorption peak located at 360 nm for CDs prepared from the aqueous extract of the rose flower was weaker than the CDs prepared from alcoholic extracts of the rose flower [87]. The evidence reported in the literature regarding carbon dot characterization using UV-vis spectroscopy shows that all types of carbon dots are active in the UV-vis region of the electromagnetic spectrum, and the fluorescent radiation of carbon dots exhibits λ_ex_-dependent behaviour. 

### 3.2. FTIR Measurement

**Figure 4:**
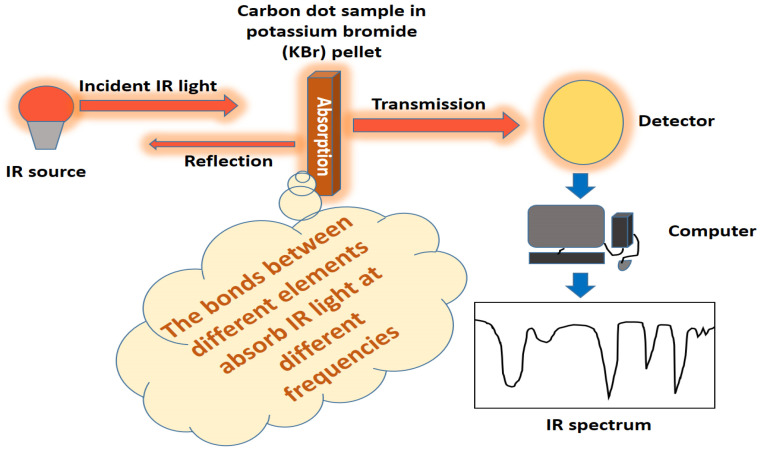
The basic setup for the measurement of IR spectra.

Singh et al. [15] reported that CQDs usually compromise carbon, hydrogen, nitrogen and oxygen. The surface of CQDs is usually comprised of various functional groups, including hydroxyl groups, carboxyl groups, carbonyl groups as well as ether or epoxy, depending on various synthesis techniques [15]. Therefore, FTIR can be performed to determine the surface functional groups of CQDs [106]. The fundamental theory of FTIR analysis is that the bonds between different elements absorb light at different frequencies. Subsequently, with FTIR analysis, the light is measured using an infrared spectrometer, which produces the output of an infrared spectrum (Figure 4). For example, the peaks appearing in FTIR spectra of CDs prepared by ultrasonication irradiation of crab shells were at 3398 cm^−1^, 2930 cm^−1^, 1640 cm^−1^, 1563 cm^−1^ and 1415 cm^−1^, which correspond to the stretching vibration of -H stretching, N-H stretching, C-H stretching, C=O stretching, N-H bending and C=C stretching [25]. The C-dots produced from pipe tobacco were functionalized with O-H groups, carboxylic groups and alkyl groups, as evidenced by the peaks at 3427 cm^−1^, 1701 cm^−1^, 1517 cm^−1^, 1282 cm^−1^, 1117 cm^−1^, 2968 cm^−1^ and 2935 cm^−1^, respectively [107]. The characteristic absorption peaks at 1724 cm^−1^ and 3307 cm^−1^ confirmed the existence of carboxyl groups on the surface of CDs, which developed through the compound oxidation of carbon strands [108]. The peaks that appeared in FTIR spectra of CDs at 1579 cm^−1^ and 1097 cm^−1^ corresponded to the presence of a double bond and ether linkage, respectively [108]. The FTIR method’s advantages for describing surface functionalization of carbon dots are that it is affordable, easy to perform with a simple sample preparation method and rapid. However, the IR cannot give adequate structural information on carbon dots and doping with metal heteroatoms in carbon dots.

### 3.3. Electron Microscopy Approaches

Electron microscopy techniques are well used in science, material science, pharmaceuticals and other research and development areas for the characterization of nanoparticles. The application of scanning electron microscopy (SEM) and transmission electron microscopy (TEM) for CD visualization has been recognized as a primary approach by various researchers, as these can provide significant information on the morphology, particle size, crystalline organization and size distribution of C-dots [109]. In typical SEM, the images are produced by scanning the surface of the CD sample with a focused electron beam that can interact with the atoms of the CD sample and stimulate signals that contain information relating to the surface topography and composition of CDs [109]. However, TEM is more precise when the measurement of CDs exceeds the resolution of SEM, which is within the range of 1–20 nm. This is because TEM affords higher resolving power (~0.2 nm), which is more suitable to identify small-size particles compared to SEM; TEM utilizes high-energy electron beams to transmit through the CD sample to obtain images [15,109,110]. For example, an average size of 12 nm of silica-encapsulated CDs and an average size of 0.9 nm of organosilane-synthesized CDs were identified using TEM in a study conducted by Wang and colleagues [111]. Dager et al. [101] also employed the TEM technique to identify the size of CQDs synthesized via microwave irradiation of Fenugreek seeds, where the CQDs obtained had an average diameter of 4.25 ± 0.56 nm. Recently, high-resolution TEM (HRTEM) has been extensively and successfully applied by various scientists for CD structure analysis and lattice imperfections [109]. HRTEM has also been applied to analyse the periodicity of the graphite core and the crystalline organization of CDs [6]. The average size of 3.4 nm of CDs was identified by Cheng et al. [44] via the HRTEM technique. The HRTEM images obtained also indicated that the CD particles prepared by Cheng et al. [44] displayed in the form of quasi-zero dimension and high crystalline, with a size distribution in the range of 1 and 10 nm. Another typical experiment also utilized HRTEM to characterize CDs prepared from tomatoes. The study stated that the as-prepared CDs were spherical in shape and had a size distribution of 5 to 10 nm [20]. The nondestructive electron microscopic technique is the only technique that can directly observe and measure the individual morphology and size of a nanoparticle. 

### 3.4. XRD

**Figure 5:**
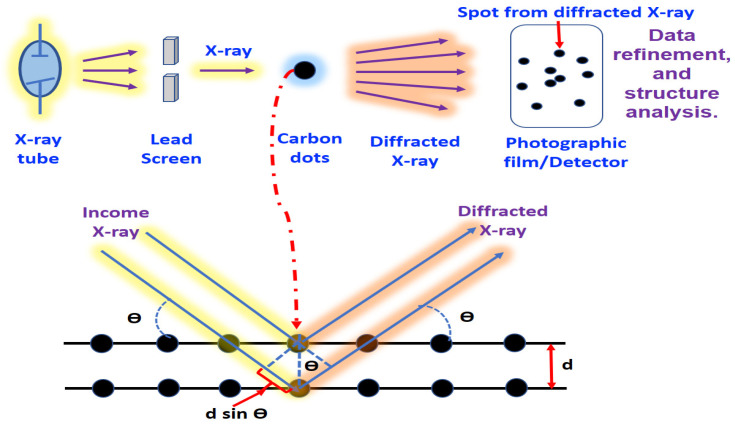
The graphic demonstration of X-ray diffraction. The variable d indicates the distance between the atomic layers, and θ indicates the angle of incidence and scattering X-ray beam (Bragg law).

XRD is another important structural tool that is efficiently used to characterize CDs, as it can provide important information regarding the particle size and phase purity as well as investigate the crystalline nature of CDs [6,15]. XRD is also able to evaluate the crystal spacing within the crystalline carbon cores of CDs and their corresponding unit cell dimensions [15]. When the monochromatic X-rays interact with a carbon dot sample, constructive interference is produced. The electrons will be excited with X-rays, creating a diffraction pattern that renders the regular spatial arrangement. These diffracted X-rays are then detected, processed and counted, revealing the average structure of nanomaterials (Figure 5). 

The diffraction pattern obtained is unique and can be used as a fingerprint of periodic atomic arrangement, which can be determined by the distribution of atoms within the lattice [112]. Bandi’s team synthesized CDs from onion waste using the hydrothermal synthesis procedure. The structures and optical properties of the as-prepared CDs were examined. The XRD diffractogram demonstrated a diffraction peak at 2θ = 22.79°, which was ascribed to the graphitic carbon (002) diffraction pattern, where the larger interlayer spacing *d* for the corresponding peak (*d* value = 0.39 nm) obtained when compared with graphite (*d* value = 0.34) was mainly attributed to the presence of oxygen-containing functional group [70]. Arul and Sethuraman [76] isolated CDs through hydrothermal treatment of *Actinidia deliciosa* (kiwi) fruit extract; the related XRD design showed an intensive broad peak around 2θ = 28.5° and a weak peak at 2θ = 40.3°, which correlated to the graphite carbon (002) and (001) diffraction patterns [76]. The Scherer formula (D = kλ/βcos) was then applied to calculate crystal particle sizes by selecting the highest peak value shown in the XRD diffractogram, where the k refers to the shape factor, which takes the value of about 0.9, λ refers to the X-ray wavelength (CuKα radiation is equal to 0.154 nm) and β refers to the diffraction peak at half of the observed maximum intensity (FWHM) [113]. Thus, according to the Scherer formula, the broad peak obtained in the XRD pattern indicated the formation of CDs with small particle sizes. Even though XRD is an essential technique for determining the critical features of C-dots with a crystallite structure, it is not appropriate for characterizing amorphous C-dots. 

### 3.5. Zeta Potential 

**Figure 6:**
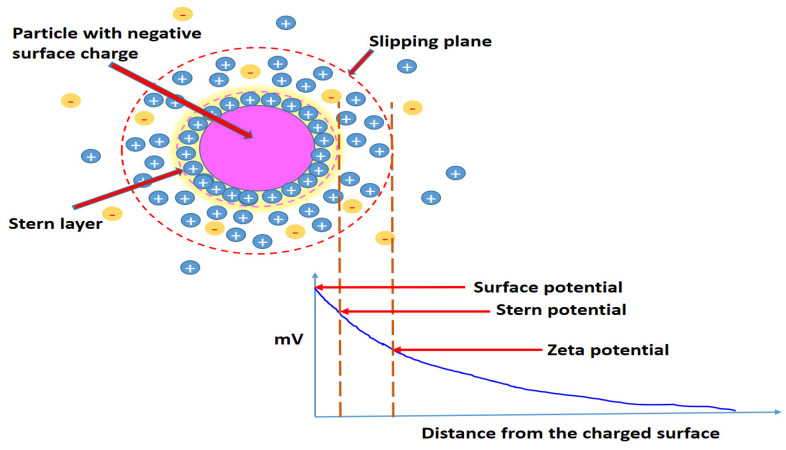
Schematic representation of zeta potential of a nanoparticle.

The zeta potential is another essential measurement to evaluate the effective electric charge on the surface of nanoparticles and quantify the charges [114]. It is an important measurement to analyse the stability of the colloidal system (probe) and the surface effects of nanoparticles, as it affects the toxicity of nanoparticles as well as the initial absorption of nanoparticles onto the cell membrane [115]. Zeta potential is the electrical potential in the interfacial double layer at the location of the slipping plane, as shown in Figure 6. The zeta potential is measured as the potential difference between the dispersion medium and the stationary layer of the fluid attached to the particle layer (Figure 6). The zeta potential’s magnitude indicates the colloidal system’s potential stability. According to Sivasankaran et al. [116], a higher value of zeta potential indicates the stability of the system, whereas the positive and negative sign of the zeta potential indicate the surface charges of nanoparticles, where nanoparticles with low zeta potential value will aggregate. For example, nanoparticles with zeta potentials larger than +30 mV are considered as strongly cationic, nanomaterials with zeta potentials value ranging from −10 to +10 mV are seen as neutral, and nanomaterials with zeta potentials less than −30 mV are expressed as strongly anionic [117]. Sachdev and Gopinath [118] isolated CDs from coriander leaves by hydrothermal approaches. Zeta potential analysis was evaluated using a zeta potential analyser, and a negative zeta potential value (−24.9 mV) was obtained, which was mainly associated with the presence of oxygen-containing functional groups (i.e., hydroxyl and carboxylic groups) on the surface of CDs [118]. Ramanan’s group synthesized CDs from algal bloom through microwave irradiation and successfully obtained a highly negative zeta potential value (−22.3 ± 8.39 mV), further indicating that synthesized CDs are negatively charged and rich in carboxyl functional group [119]. The zeta potential measurement of a carbon dot provides valuable insight into the stability and aggregation of the carbon dot.

### 3.6. Quantum Yield Analysis

**Figure 7:**
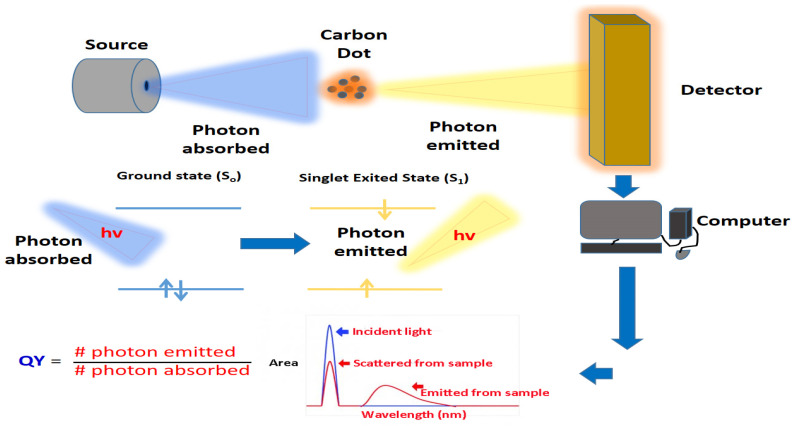
Schematic representation of fluorescent quantum yield determination.

The luminescence quantum yield (QY) is one of the important photophysical parameters that characterize the emission performance of CDs (Figure 7). In general, QY refers to the efficiency of converting absorbed light into emitted light, which can be in the form of fluorescence [120]. Even at low concentrations, high QY fluorophores often emit strong fluorescence. Therefore, high-QY CDs usually have a wide range of applications in biomedical, optical and energy fields [3]. Usually, the QY of CDs can be determined by a relative method using quinine sulphate in 0.10 M of H_2_SO_4_ solution or fluorescein in 0.10 mol/L NaOH as a reference standard [121].

The QY of CDs can be calculated by the following equation: $Q_{x}=Qs\;\frac{E_{s}}{E_{x}}\frac{I_{x}}{I_{s}}\frac{n_{x}^{2}}{n_{s}^{2}}$. where *Q* is the quantum yield, *E* is the optical density, *I* is the measured integrated emission intensity and *n* is the refractive index (1.33 for water). The subscripts *s* and *x* are the reference standard and the testing sample (CDs solution), respectively [122]. 

According to the reported studies, most of the bare CDs have QY below 10% [120]. However, doping surface functionalization of CDs can improve their QY. As an example, Zhou et al. [36] reported that the fluorescence quantum yield of CDs synthesized by a simple carbonization method using watermelon peel as a carbon precursor was about 7.1% by measuring against an aqueous solution of quinine sulphate. On the other hand, Parvin and Mandal [123] reported a QY of about 74.16% for N-doped CQDs synthesized by the hydrothermal route, using agarose as starting materials. In addition, a study conducted by Liao et al. [124] also clearly reported a significantly enhanced QY effect for N-doped CDs (28.46%) compared to non-doped CDs (5.31%).

The fluorescent quantum yield of carbon dots is essential to judge whether carbon dots can act as efficient luminous materials for their practical applications. In addition, the quantum yield is one of the key indicators used to evaluate carbon dots’ optical performance, such as in cell imaging. The higher the quantum yield value, the better the imaging effect.

## 4. Properties of Carbon Dots

Normally, CQDs have an optical absorption in the UV region with a tail extending to the visible range [14]. Most CDs possess an absorption band of around 260–323 nm, no matter how they are synthesized [9]. Yet, there may be some absorption shoulders in the absorption spectra that associate with the π–π* transition of the C=C bonds or n–π* transition of the C=O bonds of CDs. The surface passivation of CQDs with various molecules has been proven to affect CD absorption and result in a shift of absorbance to a longer wavelength [9].

### 4.1. Photoluminescence (PL)

One of the most notable features of CQDs is the excitation-dependent PL, also referred to as excitation-dependent fluorescent emission [6]. In general, a feature of the PL for CQDs is the district λ_ex_ reliance on the emission wavelength and intensity [14]. This was addressed by a study conducted by Zhang’s group, who explored the emission behaviour of CQDs with different concentrations under irradiation at 470 nm [125]. They found that the PL intensity of the yellow-emitting CQDs was firstly increased to a maximum λ_ex_ and then decreased when the excitation wavelength and the concentration of CDs were increased [125]. This experiment proved that the excitation-dependent PL behaviour of CDs is similar to other luminescent carbon nanoparticles [125]. This phenomenon might be due to the difference of emissive traps on the CQD surface or the optical selection of nanoparticles with different sizes (quantum effects), or another mechanism that is currently unresolved [126]. Hence, future research can be conducted to further clarify the exact mechanism of PL. The variation of sizes of CQDs, their wide spectra range and the intensity of the emission peaks underline their multicolour properties. CD emission colour can be tuned in line with the excitation wavelength; this exclusive property found in CQDs has been extensively used in varied applications. Pan et al. [127] also proved that the multicolour emission of fluorescence CDs depends on the excitation wavelength, via single-particle fluorescence imaging experiments, where MCF-7 cells were treated with CDs. In this case, different colours of emission in MCF-7 cells were observed under the excitation light of 405 nm, 488 nm and 543 nm [127]. Early research has shown that the luminescence property of CQDs could be achieved via a post-synthesis chemical reaction, also known as surface passivation after the surface functionalization of carbon nanoparticles [6]. Yet, CDs that exhibit single emission wavelength do not exhibit any excitation-dependence luminescence properties and can be synthesized through CD surface modification, whereby the elimination or reduction of the concentration of surface defects underlines the energy status of the carbon nanoparticles [6]. For example, Sun et al. [4] reported the presence of surface energy traps that turn into emission through surface passivation associated with the PL properties of CQDs, where the quantum confinement impact of emissive energy can be trapped on the surface of the CQDs and cause them to achieve strong PL emission. Consequently, in addition to the different sizes of CQDs, the emissive trap sites that are dispersed on the surface of CQDs will also lead to the multicolour emission of CDs [6]. Modulation of CDs’ structural or functional properties through chemical means were also proven to control the excitation-dependent profile of CDs. One of the experiments performed by Baruah, Deka and Chowdhury [128] showed a fascinating example of the close relationship between CD luminescence and surface functional groups. They demonstrated a simple and reversible on–off fluorescence switching by functionalization of CDs through an esterification reaction. The CDs synthesized from citric acid (carbon precursor) exhibit pH-dependent PL, where diminished fluorescence can be observed in highly acidic conditions (pH 1) [128]. Nonetheless, the esterification of carboxyl and hydroxyl group-bearing CDs with alcohols results in the production of strong fluorescence CDs [128].

### 4.2. Electrochemical Luminescence (ECL)

ECL is a parameter that is commonly applied to explore the morphology, composition and surface structure of CQDs [9,14]. According to Jelinek [6], ECL processes appear during the excitation of electrons and holes in the target molecules, i.e., CDs. This then subsequently leads to the relaxation of CDs to their ground states simultaneously after the light emission [6]. Commonly, CDs are synthesized via the oxidation of carbon precursors such as natural resources, graphite and saccharide. According to Zheng et al. [129], CDs that are synthesized usually exist in high oxidation form; the oxidation does not expedite the emission, whereas the reduction of CDs can enhance the luminescence. The ECL of CDs was expressed by Zheng et al. [130] through the electrochemical processing of graphite. In principle, the surface states of CDs play an important role in the formation of two states of CQDs, which are the oxidized (R^+^) and reduced (R^−^) states [9]. If the R^+^ is more unstable than R^-^, this is usually due to the cathodic ECL strength being lower than the anodic one. The electron-transfer eradication of the two oppositely charged carriers (R_+_ and R^−^) created the excited state (R*) of CQDs, whereby these R* stated CQDs later emitted a photon and returned to the ground state through light emission [9]. In particular, CQDs with low oxidation levels are denoted as r-CQDs, whereas CQDs with high oxidation levels are designated as o-CQDs; they can be synthesized through a carbonization–extraction and carbonization–oxidation technique, respectively [9,14,131]. It is valuable to know that the electrochemical reaction is determined by the dispersion of o-CQDs onto the surface of the electrode; the direct oxidization of o-CQDs will also affect the ECL emission [14]. According to Wang and Hu [14], the ECL emission corresponds to the direct oxidation of CDs. This is because the ECL wave that starts at 1.10 V and reaches its peak at 1.30 V is consistent with the oxidation peak in the cyclic voltammograms (CVs) [14]. Moreover, ECL sensing has recently been recommended by researchers as a result of stable ECL response over time. Zheng and colleagues reported that intense ECL emission was identified from carbon nanocrystals synthesized from electrochemical approaches when the potential was cycled between +1.8 and −1.5 V [130]. 

### 4.3. Phosphorescence

Phosphorescence has shown wide potential applications in the biomedical area, including bio-imaging and fluorescence sensors for ion detection, due to its large stokes, high signal-to-noise ratio and long-lived luminescence properties in nature [132]. CDs that show strong fluorescence effects also possess a minimal phosphorescence at room temperature [14]. For example, an ultralong phosphorescence lifetime was also achieved from CDs and composite matrices, as the lifetime of its phosphorescence was prolonged to the sub-second order (380 ms) [132]. The phosphorescent CDs also have received increasing attention from various researchers due to their good potential as candidates for room-temperature phosphorescent (RTP) material to replace common RTP materials such as oxides, sulphides and rare earth fluorescent materials, which are toxic, expensive and difficult to prepare [133]. CDs that acquire significance in biomedicine applications further boost the exploration of their practical applications (Table 1, Table 2, Table 3, Table 4 and Table 5). Accordingly, a pure organic RTP can be prepared by dispersing water-soluble CQDs into certain matrixes, such as polyurethane or polyvinyl alcohol (PVA), and exciting with UV light [132]. Preliminary research has shown that the phosphorescent features of CDs originated from the triplet excited states of aromatic carbonyl groups, which can be found on the CDs surface. However, the triplet states of CDs can be simply altered by the external surrounding which led to phosphorescence quenching [133]. In this case, the matrix PVA molecules proved that they could prevent the loss of the triplet excited state energy effectively from vibrational and rotational processes via embedding the groups with hydrogen bonding temperature [14]. Nonetheless, there are still some challenges in developing CD phosphorescence. In this case, the fluorescence mechanism of CDs can be further extended and exploited to have a better understanding of the CD phosphorescence properties as well as develop more alternative methods for phosphorescence CD preparation.

### 4.4. Chemical Luminescence (CL)

CL properties of CDs were first discovered by Lin et al. [134] when CDs coexisted with classical oxidants, including potassium permanganate (KMnO_4_) and cerium (IV). In principle, the oxidants generate electrons and holes into CDs, resulting in the release of energy in the form of CL emission [6,14]. When electrons are injected, the subsequent fluorescence will occur during the electronic transition from the excited energy level to a lower level [6]. The degree of CL depends on the concentration of CQDs, the yield and occurrence of the redox reactions that are responsible for the hole or electron transfer and the efficiency of coupling between these reactions [6,14]. The CL intensity is also dependent on the temperature, where an increase in temperature will lead to a beneficial effect on CL emission, which is consequently due to the thermal equilibrium of electron distribution in CDs [14]. CL in CDs has been used to detect various analytes through the participation of CDs in the oxidation or reduction reactions that contribute to the formation of electron holes [14]. It is surprising to find that the CL features can be produced by modifying CDs’ surface groups [135]. Dou et al. [136] reported a unique CL phenomenon discovered from the CQDs that are synthesized from the concentrated sodium hydroxide (NaOH) solution. This study showed the electron-donating ability of CQDs against dissolving oxygen to generate superoxide anion radicals (O_2_^-^) in a strongly alkaline solution [136]. Taken together, the CQDs’ CL properties provide new insight for research on reductive substances as well as enable the potential application of CQDs in biomedical fields such as optronics and catalysis. 

### 4.5. Up-Conversion Photoluminescence (UCPL)

UCPL is an interesting optical feature of CDs and is mainly associated with multi-photon activation, which is involved the simultaneous absorption of two or more photons and results in the emission of light with a shorter wavelength than excitation light [6,14]. The CQDs UCPL feature opens a new window for various scientists to explore applications in energy and bioscience technology, such as cellular imaging using two-photon luminescence microscopy and highly capable catalyst design [14]. The reported study illustrated that CDs also show strong fluorescence with two-photon excitation in NIR region [137]. It was found that CQDs showed a great emission effect in the visible region when excited by an argon-ion laser (458 nm) or by the femtosecond pulsed laser for two-photon excitation in the NIR range (800 nm) [137]. The representative two-photon luminescence spectrum proved the UCPL properties of such CDs. However, a recent investigation carried out by Wen et al. [138] proved the lack of UCPL characteristics in five differently prepared CQDs. They explained that the UCPL property of CQDs originated from the excitation of normal fluorescence by the leaking component from the second diffraction in the monochromator of the FL spectrophotometer [138]. The leaking component, and thus the UCPL, can be removed by inserting a long-pass filter in the excitation pathway of the fluorescence spectrophotometer [138]. The experiment also revealed that UCPL has normal fluorescence with a linear reaction instead of multiple phonon processes, which observable UCPL may not possess in most CQDs. Hence, the elimination of normal fluorescence is a must when observing the UCPL in CQDs [9,14,138]. 

### 4.6. Photoinduced Electron Transfer (PET)

The use of PL in the conversion of light energy or other relevant regions has attracted interest from various researchers to explore the photo-response of CDs and the separation of photoinduced charge as well as the process of electron transfer. According to Li et al. [126], the PL from CDs can be extinguished efficiently by electron donors or electron acceptor molecules in a solution. For example, Wang et al. [139] found that PL from CDs in an aqueous or organic solution can efficiently be quenched by the existence of electron donors such as N,N-diethylanilineor and electron acceptors such as 4-nitrotoluene and 2,4-dinitrotoluene. Particularly, photoexcited CDs are exemplary electron acceptors and donors [139]. They also state that PL can efficiently be quenched with CQDs by surface-doped metals via the excited state redox reaction interruption [139]. Yu et al. [140] investigated the transformation of the electron in the nanocomposites of carbon nanodots (CNDs), including carbon CNDs-graphene oxide (GO), CNDs-multiwalled carbon nanotubes (MWNTs) and CND-TiO_2_ nanoparticles without linker molecules, and found that fluorescence quenching can be observed significantly in CNDs-GO systems. This was mainly associated with the ultrafast electron transformation process from CDs to GO with a time constant of 400 fs [140]. Hence, this study proved that the inclusion of carbon nanotubes results in the formation of static quenching in fluorescence CDs, whereas no changes were detected in CQD–TiO_2_ and CND–MWNT nanocomposites [140]. This photo-induced electron transfer (PET) property of CQDs that can serve as an electron acceptor or electron donor may provide new insight into exploring the CQD application in catalysis and light energy conversion, as well as mechanistic elucidation. 

### 4.7. Cytotoxicity of CQDs 

Cytotoxicity refers to the capability of particular chemicals or mediators to induce cell apoptosis (programmed cell death) or necrosis (accidental cell death) to destroy living cells [15]. Numerous investigations have been performed to explore the possible applications of CQDs, especially in bioimaging and cell imaging. In this case, biocompatibility is the most decisive property that influences the possibility of CQDs replacing organic dyes and metallic quantum, which are commonly used in bioimaging and cell imaging [15]. Hence, CQD cytotoxicity should be taken into consideration by researchers. In a typical experiment, Edison et al. [141] explored the CDs’ cytotoxicity effect on Madin-Darby kidney (MDCK) cells and HeLa cells. The as-prepared CDs showed very low cytotoxicity in both cells line even though treated with high concentration. The obtained outcomes showed the good biocompatibility, low cytotoxicity and good biosafety features of CDs, which can be applied as staining probes in cell imaging applications [141]. Pal, Mohiyuddin and Packirisamy [142] performed in vitro and in vivo cytotoxicity investigation of CDs synthesized from curcumin via hydrothermal treatment. Cell viability assay was conducted to explore the biocompatibility of CDs towards human colorectal adenocarcinoma (HCT-15), human lung adenocarcinoma cells (A549) and mouse embryo fibroblast cells (NIT 3T3) [142]. The research was further expanded to the in vivo level, utilizing zebrafish embryos as a model system. The evaluated in vitro and in vivo experimental results showed CDs possessed good labelling potential and less cytotoxicity [142]. All the above experiments suggested the adequacy of CDs for in vitro as well as in vivo imaging and drug delivery studies, as they possess good biocompatibility properties that further indicate the possible application of CDs as optical imaging agents to replace common United States Food and Drug Administration (FDA)-approved organic dyes such as indocyanine green [9,15]. However, literature searches showed that only limited studies have been reported to examine the cytotoxicity of CDs; thus, more studies should be performed to support the utilization of CDs in drug delivery and bioimaging studies.

## 5. Biomedical Application of Carbon Dots

**Figure 8:**
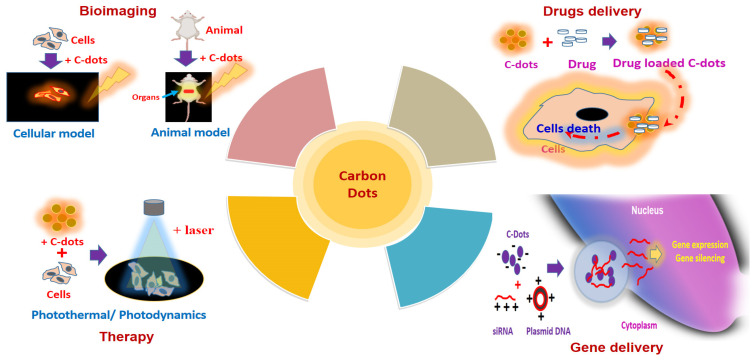
Various biomedical applications of carbon dots.

CDs, which are a new category of carbon nanoparticles that consist of quasi-spherical, discrete fluorescent carbon nanomaterials with a diameter of less than 10 nm, have multiple advantages over semiconductor QDs, including high water solubility, low cost, excellent biocompatibility, chemically inertness, highly tunable photoluminescence and electrochemical luminescence [7,143]. Because of their unique properties, CQDs have acquired significance in nano-chemistry, which has resulted in the discovery of CDD applications, especially in biomedical applications (Figure 8). As evidenced in Table 1, Table 2, Table 3, Table 4 and Table 5, CDs that were produced via various techniques have been used in numerous applications, including bioimaging, biosensing, chemical or fluorescence sensing, nanomedicine, electrocatalysis and photocatalysis; a summary of advantages and limitations of biomedical applications of CDs is given in Table 6.

Carbon dots, with exclusive optical properties, huge surface area, multicolour emission profile, small sizes, low cytotoxicity, superior biocompatibility, and excellent photostability, make them an ideal nominee for biomedical applications.

### 5.1. CDs in Bioimaging 

Bioimaging technology requires the assistance of technology, including X-ray, ultrasound and magnetic resonance imaging, to process images of living organisms [15]. Properties such as low toxicity, biocompatibility and fluorescent properties make CDs more favourable in the visualization of the biological system both in vitro and in vivo [15]. For instance, Ray’s team successfully synthesized water-soluble fluorescent CDs with a diameter between 2 to 6 nm by nitric acid oxidation of carbon soot. The synthesized CDs with green fluorescence under UV exposure showed promising applications in cell imaging, as they can penetrate HepG2 cells beyond any further functionalization [144]. Carbon is generally non-toxic and green; thus, carbon cores of CDs are also non-toxic. It has been demonstrated that the cytotoxicity effect mainly originates from the passivating agent on the surface of CDs. Therefore, surface passivating chemicals with low toxicity can be utilized securely for in vivo imaging at high concentrations [5,15]. In a typical in vivo experiment, the cytotoxicity and in vivo toxicity of the oligomeric polyethylene glycol (PEG_1500N_)-dopped CQDs were investigated by Yang and colleagues. This 28-day in vivo experiment involved intravenous injection of PEGylated CQDs into rats (8–40 mg/kg), proving that these PEGylated CQDs did not show any noticeable toxic effect in rats for toxicity assessment [145]. Physiological indicators were at the same level when the rats were exposed to different CQD concentrations and the control (0.9% NaCl only). The dissected liver and spleen of mice also showed no abnormalities, even though the dosage of CQDs presented in the harvested organs was greater than those presented in other organs [145]. Accordingly, this study proved the non-cytotoxicity and non-toxicity effect of CQDs at various concentrations and times that can be applied for in vivo imaging performance. In addition, in vitro research carried out by Ding et al. [146] found that the DNA-CQDs isolated from bacteria DNA were internalized by human embryonic kidney cell lines (HEK 293). The fluorescent DNA-CDs, which can be easily imaged by using a confocal microscope, suggest the promising application of DNA-CDs as a luminescent vehicle for cell imaging study [146]. Another study conducted by Zheng’s group showed the CQDs possess excellent biocompatibility and highly tunable photoluminescence and can easily pass through the blood–brain barrier and target the C6 glioma cells accurately without the aid of any other targeting molecules [147]. This indicated the potential application of CQDs in fluorescent imaging, with integration in the construction of CQDs as intelligent nanomedicine. 

### 5.2. CDs in Biosensing and Chemical Sensing 

**Figure 9:**
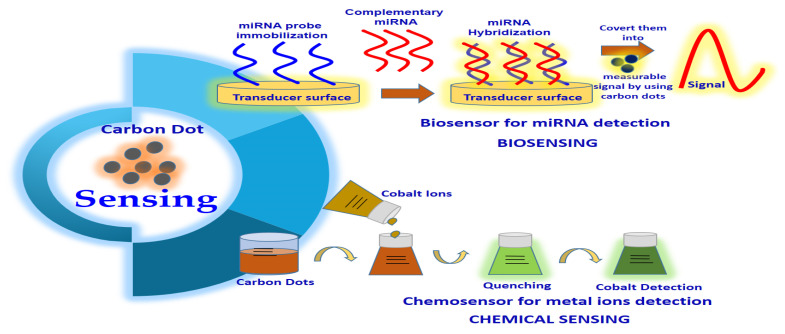
Carbon dot application in sensing.

CDs also have a wide application in biosensing and chemical sensing (Figure 9) due to characteristics such as excitation-dependent emission, biocompatibility, low cytotoxicity, water solubility as well as higher photostability [13]. The biosensors established from CDs might be utilized to examine numerous parameters or materials such as cellular ions, antibodies, proteins and nucleic acids [9]. For example, Yu’s team suggested that CQDs can form a fluorescence resonance energy transfer (FRET) system with organic dyes, which can serve as a highly sensitive radiometric sensor that can penetrate the cells easily to detect intracellular H_2_S levels. In this case, the CQD–organic dye conjugates, which emit blue fluorescence, will convert to green in the sight of H_2_S [148]. Yu’s team also expressed that the alteration of H_2_S physiological levels inside the HeLa and murine aneuploid fibrosarcoma cell line (L929) can be detected via CQD–organic dye conjugates under a fluorescence microscope. This is because a change in emission colour from blue to green was detected as a consequence of the exposure of cells to H_2_S [148]. This study provided new insight into the potential application of CQDs as detecting analytes in human health diagnosis [148]. CQDs can be utilized as fluorescent labels in immunoassays. For example, Posthuma-Trumpie et al. [149] reported that CQDs can act as signalling labels for the detection of biomolecules, including antibodies, nucleic acid and proteins. CQDs can also be employed as an impressive fluorescence sensor in the detection of nucleic acid. The cationically modified CQD probes that were synthesized from Han’s group proved that these CQD probes were able to emit spectrally detectable fluorescence when bound with double-stranded DNA as well as single-stranded RNA in living cells [150]. Interestingly, these CQD probes can pass through various biological barriers in vitro and in vivo [150]. This study also provides a new platform to study the CQD application by exploring the localization and motion of DNA and RNA in living cells. 

CDs are promising candidates for chemical sensing or fluorescence sensing. The interaction of metal ions and the surface functional groups of CQDs will result in the production of new electron-hole recombination and lead to a change in the fluorescent nature of CDs with the aid of the energy transfer route [13]. In this case, CDs can be utilized as a fluorescence sensor for heavy metal ion detection in aqueous solutions or living cells, which is evidenced by numerous reported studies as listed in Table 1, Table 2, Table 3, Table 4 and Table 5. In a typical experiment, Zhou and colleagues demonstrated the use of unmodified CQDs as fluorescence-sensing agents to detect highly toxic metal ions (Hg^2+^ ions) and biothiols (e.g., cysteine, homocysteine, glutathione) [151]. They found that Hg^2+^ was able to quench the fluorescence of the CQDs, whereas biothiols can efficiently prevent fluorescence quenching because of their ability to remove Hg^2+^ from the surface of CQDs [151]. Other applications of CDs in chemical sensing included Fe^3+^, Cu^2+^, Cr(VI), Ag^+^ and Pb^2+^ detection due to the formation of fluorescent quenching of CQDs with these heavy metal ions. For instance, Liu and colleagues designed a highly sensitive lysine (Lys)-enhancing CD-BSA fluorescent probe to detect Cu^2+^ in tap water and hair [152]. They found that Cu^2+^ was able to react with the –COOH and –NH_2_ functional group on the surface of the Lys-enhancing CD-BSA fluorescent probe, causing fluorescent quenching [152]. CDs possessing excellent electro-chemiluminescence and chemiluminescence also led to the development of the use of CDs in electro-chemiluminescent assay and chemiluminescent assay for the detection of various ions and compounds, such as Co^+2^, NO_2_^−^ and pentachlorophenol [5]. 

### 5.3. CDs in Photocatalysis 

One of the most predominant and exciting topics in nanoscience and nanochemistry is nano-photocatalysis [9,126]. Recently, various studies were conducted in the discovery of new and more potent nanocatalysts with excellent specificity, high selectivity and strong and tunable chemical activity. In this case, CQDs that exhibit the potentiality of harnessing long-wavelength light and vitality conversion provide the possibility for application as photocatalysts in organic synthesis [5,9]. In fact, Ma’s group indicated that the water-soluble fluorescent CQDs synthesized via simple ultrasonic reaction showed incredible catalytic activity to decompose H_2_O_2_ and also NIR light-determined electron transfer activity [153]. In this experiment, CQDs with sizes of 1–4 nm acted as an effective NIR light-determined photocatalyst that could selectively oxidize alcohols to benzaldehyde; the photocatalytic action of CQDs could be efficiently regulated by the doping of CQDs as well as modifying the CQDs surface [5,15,153]. TiO_2_ is one of the most well-known photocatalysts and is commonly used to eliminate organic pollutants as well as generate H_2_ by splitting of water [154]. Nonetheless, a noteworthy disadvantage of TiO_2_ photocatalytic efficiency is the impotent use of visible light as the illumination source. Hence, bandgap construction by possible adjustment of TiO_2_-based media can be done to enhance the execution of TiO_2_ catalysts [5,15,154]. In this case, a nanocomposite of CQDs and TiO_2_ is recommended to understand the effective usage of the full sunlight spectrum [5,15]. For example, Li’s team designed a TiO_2_/CQDs complex system to exploit the employment of sunlight with a full spectrum [155]. They found that the TiO_2_/CQDs complex was able to deteriorate methylene blue (MB) under visible light illumination, whereas the control groups, which used only pure TiO_2_ without CQDs, showed no or little reduction of MB [155]. The result proved that CQDs are essential for effective photodegradation under visible light [155]. The above studies provide a new approach to the utilization of CQD-designed photocatalysts in bioscience and energy technology.

### 5.4. CDs in Nanomedicine (Photodynamic, Photothermal, Drug Delivery Applications)

One of the most interesting applications of CDs is nanomedicine. CDs, which are a type of fluorescent nanoparticles, show no toxicity under in vitro and in vivo experiments. In a typical experiment, Singh’s team performed in vitro and in vivo toxicity studies of nitrogen-doped CQDs (NCQDs). In a typical in vitro experiment, lactate dehydrogenase (LDH) profile, cell apoptosis analysis, DNA fragmentation and growth cycle assessment was analysed by treating the HeLa cell line with NCQDs [156]. The results showed no apparent toxicity of NCQDs against these cancer cell lines. Singh et al. [156], also in an in vivo toxicity study, treated mice with two different concentrations of NCQDs for 30 days. The antioxidant, serum biochemical, haematological and histopathological analysis indicated that CQDs did not show any noticeable toxicity towards mice at both NCQD concentrations [156]. Hence, CQDs are safe enough to be used in nanomedicine.

Photothermal therapy (PTT), which offers numerous advantages in conventional cancer therapy methods such as chemotherapy, radiotherapy and surgery, has attracted attention from researchers in studying its application in the cancer field [13]. PTT usually employs photothermal agents (PAs), including carbon nanomaterials, silver nanomaterials, gold nanomaterials and germanium nanocrystals, which can absorb light (preferable to the NIR region) strongly and convert light energy into hyperthermia, which leads to the generation of local heat and the destruction of cancer cells with little adverse effect on normal cells [13,157,158]. Various studies have proven that CQDs can be used in PTT for cancer treatment. For instance, Ge et al. [159] investigated the use of CDs prepared from polythiophene phenylpropionic acid in PTT. They found that the as-prepared CQDs showed significant cytotoxicity towards HeLa cells when exposed to an NIR laser. Moreover, the high in vivo PTT efficacy of CQDs towards tumour-bearing mice without extensive toxicity proposed the possible application of CQDs in the field of cancer diagnosis and treatment as well as bioimaging in living mice [159]. Das’s group studied the photothermal ablation effect of the designed mesoporous hollow NCQDs to capture carbon spheres (HCS) in human oral cancer cells (FaDu). In this in vitro experiment, they found that NCQDs-HCS can internalize with the FaDu cells and activate a significant thermal ablation effect in the cells when exposed to a 980 nm NIR laser [157]. The excellent fluorescent property of the designed NCQDs-HCS could be used to monitor the remedial reaction during the treatment [157]. 

Photodynamic therapy (PDT) is a clinical therapy that is commonly applied for superficial tumour treatment. The photosensitizer that is used in PDT must be highly sensitive so that it can localize and accumulate at the tumour tissue specifically. The photosensitizer that is used in PDT will then absorb visible light and can give rise to the production of an excited single state followed by the transition of a long-lived triplet state that can react with oxygen molecules to produce ROS, destroying cancer cells effectively [13,160]. CDS are exclusively used as a photosensitizer in PDT because of their unique and beneficial properties, such as excellent biocompatibility, low-cost synthesis and ability to conduct light energy to heat. For example, Beack et al. [161] suggested the use of transdermal CDs-chlorine e6-hyaluronate (CDs-Ce6-HA) conjugate in PDT of melanoma skin cancer. In this experiment, the CDs-Ce6-HA synthesized from the coupling response of diamino-hexane modified HA and the carboxylic group of Ce6 generated singlet oxygen compared to free Ce6. These synthesized CDs-Ce6-HA conjugates were able to target the B16F10 melanoma cells in a tumour model in mice when observed under a confocal microscope and two-photon microscope [161]. Complete suppression of melanoma skin cancers was also observed in this experiment after transdermal treatment using CDs-Ce6-HA conjugate [161]. Another study conducted by Guo’s team proved that the as-prepared Cu and N co-doped CDs can inhibit the growth of B16 melanoma cells significantly by both PTT and PDT [162]. In addition to PTT and PDT, CQDs can also be applied in radiotherapy. Kleinauskas and colleagues described that the coating of the silver shell (C-Ag-PEG CQDs) on PEGylated CQDs can be utilized as a radiosensitizer in prostate adenocarcinoma cell lines (Du145 cells). C-Ag-PEG CQD that accumulates at Du145 cells will eject electrons when exposed to low-energy X-ray and generate free radicals that can damage the cancer cells [163]. 

Recently, drug delivery systems (DDSs) depending on nanotechnology have been widely investigated. Unfortunately, various nanomaterials that can act as drug delivery vehicles, such as graphene oxides, mesoporous silica (MS), polymeric nanoparticles and gold nanoparticles (AuNPs), have toxicity and biocompatibility issues that limit their application in clinical therapy [5,9]. For example, the fluorophore quenching ability of AuNPs makes them hard to monitor in in vivo systems [5,9]. The requirement of thiol groups for drug loading through the Au-Thiol interaction further limits their application in drug delivery [164]. This has resulted in considerable attention of researchers in discovering more promising nanomaterials to replace AuNPs. As an emerging class of luminescent nanomaterials, CDs have shown tremendous potential as drug delivery agents, as they have demonstrated excellent biocompatibility properties, high water solubility and flexibility in surface modification with other chemical molecules [5,9]. For example, Zheng and colleagues conjugated oxidized oxaliplatin (Oxa(IV)-COOH), which is a type of platinum-based anticancer pro-drug that is applied in the pharmacotherapy of metastatic colorectal cancer on the surface of CQDs by condensation response between the carboxyl group of Oxa(IV)-COOH and amino group on the CQDs’ surface through chemical coupling. Zheng et al. [165] expressed that the CQD-conjugated drug absorbed by the cancer cells by endocytosis will then release upon the reduction of Oxa(IV)-COOH to oxaliplatin(II). This further suggests the application of pro-conjugated CQDs in diagnosis, estimating the proper dosage of medicine during cancer treatment [165]. CDs can also act as nanocarriers, as they can efficiently track and deliver genes or drugs; branched polyethyleneimine CQDs can be used as a potential gene delivery agent [15]. CDs also play a crucial role in controlling the release of the drug; the loading of CDs with doxorubicin can regulate the release of the drug in HeLa cells [5,14,15,166]. In a typical example, D’souzza and colleagues suggested the as-synthesized CQDs by utilizing carrot roots as a carbon precursor that can act as nano-vehicles for the delivery of mitomycin. In this experiment, the fluorescent CQDs produced were able to interact with mitomycin effectively by hydrogen bonding; this hydrogen bonding further breaks in the extracellular microenvironment of pH 6.8 and the drug to be diffused [167]. D’souzza’s group expressed that mitomycin drug-loaded CQDs with ultra-small diameter and high biocompatibility properties exhibits] high affinity towards cancer cell membranes and facilitated high degree internalization of mitomycin-CQDs by *Bacillus subtilis.* In addition, the in vitro research results from this experiment further suggest the mitomycin-loaded CQDs nanocarrier can effectively enter the tumour cells, exhibit pH-dependent release behaviour and be biocompatible with MCF-7 cancer cells [167]. In summary, the unique optical and physiochemical properties of CQDs allow them to be utilized as an effective vehicle to deliver drugs, which further suggests their potential application in diagnosis, estimating proper dosage of medicine, customizing the drug injection time and monitoring the response during cancer treatment by tracking the fluorescence signal of CQDs. The development of a highly biocompatible and fluorescent delivery system based on fluorescent CQDs further suggests the potential application of CQDs in personal medicine, with such CQDs holding great promise for specific drug delivery with minimal toxicity and adverse effects in cancer patients. Nonetheless, CDs’ specificity to target certain states of diseases remains unclear, which limits their therapeutic application. Hence, further study is required for understanding whether CQDs can specifically target diseases as well as their possible therapeutic applications.

## 6. Research gaps on CDs

The challenges of fluorescence green CDs include the following:

- Various methods have been reported for synthesizing carbon dots and require efficient standard synthesis techniques to be developed.
- Imperfect carbonization of the precursor molecules frequently leads to the formation of amorphous carbon, necessitating effective separation methods following the synthesis of carbon dots.
- CDs’ luminescence and electrochemical properties should be considered and should improve the quantum yield of carbon dots.
- The elimination route, degradation times and interfacial charge transfer mechanism of CDs are still unclear, as CDs are still in preliminary study.
- In vivo studies on the detection limits, specificity and sensitivity of CDs in targeting tumours, organs, or specific states of diseases still need to be conducted.
- Safety factors still need to be considered before researchers can use carbon dots for clinical purposes.

Future research on CDs includes the following:

- The development of a standardized method for the production of carbon dots.
- The development of an effective separation method to purify the carbon dot.
- Further study of the mechanism of action of carbon dot synthesis to improve carbon dots’ quantum yield, luminescence and electrochemical properties.
- More in vitro, in vivo and pre-clinical studies are needed to investigate carbon dots’ biological activity, toxicity and mechanism of action before researchers can use them for clinical purposes.

## 7. Conclusions and Future Perspectives 

This review summarised the current progress in CDs, including consolidation of the synthesis route, characterisation techniques, features and biomedical applications. The preparation of CDs can roughly be classified into two distinct groups: the “top-down” and “bottom-up” techniques. Plant-mediated green synthesis of CDs has become the predominant green chemistry approach and offers various exclusive benefits, which are well discussed in this review. In addition, various natural resources such as fruits, vegetables and organic wastes are used for the plant-mediated green synthesis of CDs using ultrasonic, chemical oxidation, carbonisation, hydrothermal, solvothermal, and microwave irradiation approaches. Meanwhile, various technologies used to characterise CQDs were also reviewed, such as the UV-vis spectroscopy method, FTIR, TEM, HRTEM, XRD and zeta potential measurement. The characterisation of CQDs is essential to understand the mechanism correlated with their properties, such as PL, ECL, phosphorescence, etc., which were also discussed in this review. No doubt there will be challenges in the improvement of quantum yield, luminescence and electrochemical performance, as well as limited information regarding the mechanism of CQD synthesis, which researchers should further consider. Future research must be established in this area to overcome these limitations. Simultaneously, the biomedical applications of CQDs in bioimaging, biosensing, chemical sensing, photocatalysis and nanomedicine were also reviewed. However, most of the present studies that discussed the CQDs’ biomedical applications in the literature were mainly conducted using in vitro cell lines and animal models. Accordingly, more in vitro, in vivo, and pre-clinical investigations must be conducted to assess CQD biological activity, toxicity and blood circulation properties and consolidate them into multifunctional platforms for biomedical application. Subsequently, we firmly believe the advent of more straightforward, economical and novel green synthesis routes, more promising properties of CQDs and more innovative and promising applications will be continuously uncovered for these increasingly significant carbon nanoparticles. 

## Figures and Tables

**Table 2:** The summary of CDs synthesized from different natural products by ultrasonic treatment and the respective applications.

Carbon Source	Solvents Used (Other Than Water)	Production Conditions	Application Field	References
Cigarette ash	Dimethylformamide (DMF)	30 min	Fluorescent nanomaterials	[28]
Vegetable waste	Ethanol	40 kHz/45 min/60 °C	Fluorescent sensor	[32]
Pennsylvania anthracite and Kentucky Bituminous coal	H_2_O_2_	700 W/40 kHz/5–6 h	NA	[24]
Crab shells	Folic acid	20 kHz	Cell imaging and fluorescence sensor	[25]
Soybeans	None	2 h	Fluorescence sensor	[33]
Dried *Polyalthia longifolia* leaves	None	1 h	Organic pollutant control	[26]

**Table 3:** The overview of CD synthesis from different natural carbon precursors via carbonization and the respective applications.

Carbon Precursor	Production Conditions	Application Field	References
Watermelon peel	220 °C/2 h	N/A	[36]
Citric acid	200 °C/30 min	Biosensor	[38]
Lychee seed	300 °C/2 h	Bioimaging	[39]
Chitosan	300 °C/2 h	Cell imaging	[40]
Wool	300 °C/2 h	Bioimaging and fluorescence sensor	[41]
Peanut shell	220 °C/2 h	Bioimaging	[42]
Citrus peel	180 °C/2 h	Fluorescence sensor and cell imaging	[43]
Walnut shell	250 °C/NA; 1000 °C/25 min	Bioimaging	[44]
Peanut shell	340–420 °C	Fluorescence sensor	[45]
Mangosteen pulp	10 min	Fluorescence imaging and fluorescence sensor	[46]
Date palm fronds	300 °C	Photocatalysis, bioimaging and drug delivery	[47]
*Borassus flabellifer* male flower	300 °C/2 h	Fluorescence sensor	[48]
*Setcreasea purpurea* boom	300 °C/2 h	Fluorescence sensor and fluorescence ink	[49]
Lemon juice	100 °C/45 min	Fluorescence sensor	[50]
Gynostemma	400 °C/4 h	Bioimaging	[37]
Banana peel	80 °C/12 h	Colourimetric sensor	[51]

**Table 4:** The overview of CD synthesis from various natural carbon precursors via hydrothermal treatment and the respective applications.

Carbon Precursors	Solvents Used (Other Than Water)	Production Conditions	Application Field	References
Pomelo peels	N/A	200 °C/3 h	Fluorescence sensor	[54]
Grass	N/A	180 °C/3 h	Fluorescence sensor	[62]
Cocoon silk	N/A	200 °C/72 h	N/A	[63]
Bombyx mori silk	NaOH	190 °C/3 h	N/A	[64]
Bamboo leaves	N/A	180 °C/3 h	Photocatalysis	[65]
Honey	30% H_2_O_2_	100 °C/2 h	Fluorescence sensor and cell imaging	[66]
Cabbage	N/A	140 °C/5 h	Cell imaging	[67]
Brown lentil	N/A	220 °C/7 h	Fluorescence sensor	[68]
Unripe peach fruit extract	Ammonia	180 °C/5 h	Fluorescence bioimaging and electrocatalysis	[69]
Onion waste	Ethylenediamine	120 °C/2 h	Fluorescence sensor and cell imaging	[70]
Tomato juice	N/A	150 °C/2 h	Fluorescence sensor	[55]
Lemon juice	L-arginine	200 °C/3 h	Fluorescence sensor	[71]
*Lycii fructus*	25% ammonia solution	200 °C/5 h	Fluorescence sensor and cell imaging	[72]
Pseudo-stem of banana plant	Ethanol	180 °C/2 h	Fluorescence sensor	[73]
Kelp	N/A	180 °C/5 h	Fluorescence sensor	[74]
Turmeric, lemon or grapefruit extract	Ethylenediamine	180 °C/6 h	Photoluminescence sensor	[75]
*Actinidia deliciosa* (kiwi) fruit extract	25% ammonia solution	180 °C/12 h	Catalysis, anticancer and cell imaging	[76]
*Tamarindus indica* leaves	N/A	210 °C/5 h	Fluorescence sensor	[56]
*Syringa obtataLindl*	N/A	200 °C/4 h	Fluorescence sensor, pH detection and cell imaging	[77]
*Azadirachta indica* leaves (neem leaves)	N/A	150 °C/4 h	Fluorescence sensor	[60]
Olive pits	N/A	200 °C/2 h	N/A	[57]
*Dunaliella salina*	N/A	200 °C/5 h	Fluorescence sensor and cell imaging	[78]
Sweet potato peels	N/A	200 °C/3 h	Fluorescence sensor	[79]
Rice residue	Lysine	200 °C/12 h	Fluorescence sensor	[80]
Grass	N/A	180 °C/2 h	Photocatalysis	[81]
Flowers of *Abelmoschus manihot*	N/A	220 °C/4 h	Fluorescence sensor and cell imaging	[82]
Flowers of *Osmanthus fragrans* Lour	N/A	240 °C/5 h	Fluorescence sensor and cell imaging	[83]
Dwarf banana peels	Ammonia	200 °C/4 h	Fluorescence sensor, bioimaging and fluorescence ink	[84]
Water hyacinth leaf	N/A	200 °C/4 h	Photocatalysis	[85]
Chitin	Ammonia	240 °C/10 h	Fluorescence sensor	[58]
Waste tea	Ethanediamine Cu(Ac)_2_·H_2_O	150 °C/6 h	Fluorescence sensor	[86]
Rose flower	N/A	200 °C/2 h	Fluorescence sensor	[87]
Orange peel, *Ginkgo biloba* leaves, paulownia leaves and magnolia flowers	N/A	200 °C/8 h	Fluorescence sensor	[88]
Purslane leaves	N/A	150 °C/4 h	Fluorescence sensor	[89]
*Momordica charantia* (bitter melon)	Sodium borohydride	180 °C/5 h	Fluorescence sensor	[59]
Dead leaves of *Samanea saman*	NaOH H_2_O_2_	195 ± 5 °C/16 h	Electrocatalysis	[90]
Maple leaves	N/A	190 °C/8 h	Biosensing and electrocatalysis	[91]

**Table 5:** The overview of CD synthesis from numerous natural carbon precursors via microwave irradiation and the respective application.

Carbon Precursors	Solvents Used (Other Than Water)	Synthesis Condition	Application Field	References
Silkworm chrysalis	N/A	210 °C/20 min	Cell imaging	[94]
Wool	H_2_O_2_	200 °C/60 min	Fluorescence sensor	[95]
Banana peels	N/A	500 W/20 min	Electrochemical sensor	[92]
*Bauhinia* flower	N/A	1000 W/10 min	Fluorescence sensor	[96]
Quince fruit powder	Ethanol	700 W/220 °C/30 min	Cell imaging, fluorescence sensor and drug delivery	[93]
Orange peels and banana peels	N/A	10 min	N/A	[97]
Banana peels	acetone	700 W/5 min	Colourimetric sensor	[51]
Sugarcane syrup	N/A	700 W/1.5 min	N/A	[98]
Jackfruit seeds	40% H_3_PO_4_	600 W/90 s	Fluorescence sensor and cell imaging	[99]
*Nerium oleander* ethanolic or aqueous extract	Ethanol	800 W/5–40 min	N/A	[100]
Fenugreek seeds	N/A	500 W/70 °C/5 min	Fluorescent protein crystals	[101]
Cotton linter waste	N/A	400 W/150 °C	Cancer imaging	[102]
*Gingko biloba* leaves	N/A	400–800 W/1–10 min	Photocatalysis	[103]
Sewage sludge	N/A	700 W/30 min	Fluorescence sensor	[104]
*Aloe barbadensis* Miller (aloe vera)	N/A	80 W/2.45 GHz/4–8 min	Photocatalysis and cancer cell imaging	[105]

**Table 6:** Advantages and limitations of biomedical applications of carbon dots.

Biomedical Application	Description	Analytes/Real Samples	Limitations
Bioimaging	CDs with low cytotoxicity and good biocompatibility properties can easily entered cells and distributed in the cytoplasmic region of the cells.	Cancer cells, microalgae, zebrafish, mice organs, fingerprint detection.	The elimination route of CDs remains unclear and there is still a lack of understanding of their in vivo state.
Biosensing and Chemical sensing	CDs can act as fluorescent probe for selective and sensitive detection of cellular ions, antibodies, protein and nucleic acid.	Cellular ions, antibodies, protein, nucleic acid.	The elimination route of CDs remains unclear, the degradation times of CDs are still unclear, and the understanding of their detection limits and high sensitivity for use in clinical trials is still lacking.
Photocatalysis	CDs showed high photocatalytic activity as they can decompose organic dyes, 2,4 dichlorophenol, H_2_O_2_, anionic dye, and eosin yellow under light irradiation.	Organic dyes, 2,4-DCP, anionic dye, eosin yellow.	Lack of understanding of their degradation efficiency, recombination loss and effectiveness of interfacial charge transfer.
Photodynamic therapy	CDs can be used as photosensitizer agent, as they are able to generate reactive oxygen species (ROS) to kill cancer cells when irradiated by light source.	Cancer cells	Lack of knowledge about the effectiveness for treating large, deeply hidden tumors and the doses used in clinical studies, as CDs still under preliminary study.
Photothermal therapy	CDs can be used as photothermal agent as they able to show significant cytotoxicity towards cancer cells when irradiated by light source.	Cancer cells	Lack of knowledge about the effectiveness of deeper heating of tumor tissues and thermotolerance in clinical studies as CDs still under preliminary study.
Drug delivery	CDs able to effectively track and deliver gene or drug to selected target.	Cancer cells	Lack of knowledge on the specificity of CDs to target certain states of diseases.

## Data Availability

All data generated or analysed during this study are included in this article.
